# Biochemical Markers for the Diagnosis of Mitochondrial Fatty Acid Oxidation Diseases

**DOI:** 10.3390/jcm10214855

**Published:** 2021-10-22

**Authors:** Pedro Ruiz-Sala, Luis Peña-Quintana

**Affiliations:** 1 Centro de Diagnóstico de Enfermedades Moleculares, Universidad Autónoma Madrid, CIBERER, IDIPAZ, 28049 Madrid, Spain; prsala@cbm.csic.es; 2 Pediatric Gastroenterology, Hepatology and Nutrition Unit, Mother and Child Insular University Hospital Complex, Asociación Canaria para la Investigación Pediátrica (ACIP), CIBEROBN, University Institute for Research in Biomedical and Health Sciences, University of Las Palmas de Gran Canaria, 35016 Las Palmas de Gran Canaria, Spain

**Keywords:** fatty acid β-oxidation diseases, carnitine, acylcarnitines, newborn screening, mass spectrometry, acylglycines

## Abstract

Mitochondrial fatty acid β-oxidation (FAO) contributes a large proportion to the body’s energy needs in fasting and in situations of metabolic stress. Most tissues use energy from fatty acids, particularly the heart, skeletal muscle and the liver. In the brain, ketone bodies formed from FAO in the liver are used as the main source of energy. The mitochondrial fatty acid oxidation disorders (FAODs), which include the carnitine system defects, constitute a group of diseases with several types and subtypes and with variable clinical spectrum and prognosis, from paucisymptomatic cases to more severe affectations, with a 5% rate of sudden death in childhood, and with fasting hypoketotic hypoglycemia frequently occurring. The implementation of newborn screening programs has resulted in new challenges in diagnosis, with the detection of new phenotypes as well as carriers and false positive cases. In this article, a review of the biochemical markers used for the diagnosis of FAODs is presented. The analysis of acylcarnitines by MS/MS contributes to improving the biochemical diagnosis, both in affected patients and in newborn screening, but acylglycines, organic acids, and other metabolites are also reported. Moreover, this review recommends caution, and outlines the differences in the interpretation of the biomarkers depending on age, clinical situation and types of samples or techniques.

## 1. Introduction

### 1.1. Mitochondrial Fatty Acid β-Oxidation

Fatty acid β-oxidation (FAO) of 20 carbons or less in length occurs in the mitochondria. In children after 12–24 h of fasting and in situations of metabolic stress, in which it is necessary to supply the energy of glucose once the glycogen stores have been depleted, FAO provides 80% of the body’s energy needs, mobilizing fatty acids (FAs) from adipose tissue. This process is of special importance in the first days of life, during the transition from fetal to neonate, when glucose is replaced by fatty acids as the main source of energy.

Except for the brain, most tissues use FAs, particularly the heart, skeletal muscles, and the liver. FAs are the heart’s preferred fuel after birth, and are also an important source of energy for skeletal muscles during prolonged exercise. In the liver, the oxidation of FAs provides energy for gluconeogenesis and ureagenesis. The ketone bodies formed are also carried to other tissues as auxiliary fuel. Thus, the brain uses the ketone bodies formed from FAO in the liver as the main source of energy.

More than 25 enzymes and transporters are involved in the FAO pathway and the final product is acetyl-CoA, which can be used in the synthesis of ketone bodies or in the mitochondrial electron transport chain through the Krebs cycle to form ATP.

FAO involves several stages:

1. FAs uptake by cells and activation. In order to be able to pass to the mitochondrial matrix after cell uptake, long-chain fatty acids (LCFA, C14-C20) need to be activated into acyl-CoA esters by acyl-CoA synthetase (AS), which is found in the internal phase of the outer mitochondrial membrane.

2. Cycling of carnitine to pass the FA to the mitochondrial matrix. Transport to the mitochondrial matrix requires the carnitine cycle. The four steps involved are: the entry of carnitine to the cell through the carnitine transporter (OCTN2); the action of carnitine palmitoyltransferase I (CPT I) in the outer mitochondrial membrane, which converts long-chain acyl-CoA substrates into their respective acylcarnitines; the transport of acylcarnitines through the carnitine-acylcarnitine translocase (CACT) of the inner mitochondrial membrane and, then, the re-esterification of the long-chain acylcarnitines into their corresponding acyl-CoAs by the carnitine palmitoyltransferase II (CPT II).

Carnitine can be obtained from food and also synthesized endogenously, but this synthesis, the metabolism of carnitine itself, and its associated defects, are not objective of this review.

The short-chain (<C6) and medium-chain (C6–C12) FAs pass directly to the mitochondrial matrix, without the requirement of the carnitine transport system.

3. β-Oxidation spiral. Once inside the mitochondria, the FAs enter the β-oxidation spiral. For each turn of the β-oxidation cycle, an acetyl-CoA molecule and an acyl-CoA with two carbon atoms less than the initial acyl-CoA are formed, which in turn can enter new cycles of β-oxidation as many times as necessary until only acetyl-CoA is left. β-Oxidation consists of four steps, catalyzed by:

- acyl-CoA dehydrogenase, where FAD is the coenzyme;
- enoyl-CoA hydratase;
- 3-hydroxyacyl-CoA dehydrogenase, where NAD is the coenzyme;
- 3-ketoacyl-CoA thiolase.

There are four specific FAD dehydrogenases for different acyl-CoA chain lengths:

- Very long-chain acyl-CoA dehydrogenase (VLCAD);
- Long-chain acyl-CoA dehydrogenase (LCAD);
- Medium-chain acyl-CoA dehydrogenase (MCAD);
- Short-chain acyl-CoA dehydrogenase (SCAD).

The activities of enoyl-CoA hydratase (SCEH, LCEH), 3-hydroxyacyl-CoA dehydrogenase (HAD, LCHAD) and 3-ketoacyl-CoA thiolase (S/MCKAT, LCKAT) are performed by the mitochondrial trifunctional protein (MTP), which is located on the inner mitochondrial membrane. Short-chain enoyl-CoA hydratase (SCEH, *ECHS1*) is also active in the valine catabolic pathway [1].

4. Enzymes for the oxidation of unsaturated fatty acids;

5. Electron transfer pathway. There is a continuous flow of electrons from fatty acids to coenzyme Q in the mitochondrial respiratory chain, which is mediated by electron transferring flavoprotein (ETF) and electron transferring flavoprotein dehydrogenase (ETF-QO), both of which are riboflavine-dependent;

6. Acetyl-CoA is used for the synthesis of ketone bodies.

**Figure 1:**
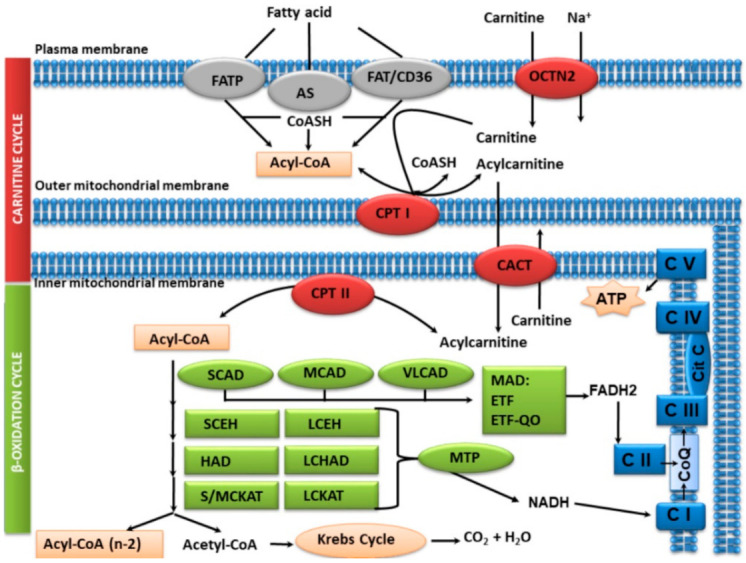
Main stages in carnitine and fatty acid ß-oxidation cycle. Uptake and activation of FA, in gray: FATP, FAT/CD36: fatty acid transporters; AS: acyl-CoA synthetase; Cycling of carnitine to pass the FA to the mitochondrial matrix, in red: OCTN2: carnitine transporter; CPT I: carnitine palmitoyltransferase I; CACT: carnitine-acylcarnitine translocase; CPT II: carnitine palmitoyltransferase II; β-Oxidation spiral, in green: SCAD: short-chain acyl-CoA dehydrogenase; MCAD: medium-chain acyl-CoA dehydrogenase; VLCAD: very long-chain acyl-CoA dehydrogenase; SCEH: short-chain enoyl-CoA hydratase; LCEH: long-chain enoyl-CoA hydratase; HAD (M/SCHAD): 3-hydroxyacyl-CoA dehydrogenase; LCHAD: long-chain 3-hydroxyacyl-CoA dehydrogenase; S/MCKAT: short/medium-chain 3-ketoacyl-CoA thiolase; LCKAT: long-chain 3-ketoacyl-CoA thiolase; MTP: mitochondrial trifunctional protein; MAD: multiple acyl-CoA dehydrogenase; Electron transfer and respiratory chain pathway, in blue: CoQ, CI, CII, CIII, CIV, CV: coenzyme Q and mitochondrial respiratory complexes I, II, III, IV and V.

The main FAO stages and proteins involved are shown in Figure 1.

### 1.2. Mitochondrial Fatty Acid β-Oxidation Diseases

After their first discussion in publication in 1972, the innate errors in the metabolism of the β-oxidation of fatty acids and of the carnitine system (FAODs) have constituted a group of diseases with several types and subtypes. FAODs are genetic disorders transmitted by autosomal recessive inheritance. Their real rate of incidence is probably still underestimated, since many cases are unperceived; therefore, this group of diseases must be treated with high diagnostic suspicion in certain clinical situations.

Since the development of newborn screening (NBS) systems, an overall incidence of 1/9300 has been proposed [2], although this varies according to program and country. NBS programs clearly prevent disabilities in diagnosed children [3], but also face significant new challenges in the diagnosis of FAODs, since new milder phenotypes are being detected, and new therapies must be proposed [4,5]. In addition, the differentiation of affected patients, carriers and false positive cases is another challenge [6,7].

The clinical spectrum and prognosis are both highly variable, depending on the enzyme deficiency and the age of the patient, from paucisymptomatic cases or cases with mild symptoms under conditions of fasting or metabolic stress, to more severe affectations. A common feature of all these disorders (except the short or sometimes medium chain) is fasting hypoketotic hypoglycemia [8,9]. Approximately 5% of sudden deaths in childhood are secondary to FAODs [10], most of which are diagnosed postmortem. With the implementation of newborn screening, the clinical presentation and its natural history have changed significantly. More detailed data regarding the clinical manifestations of each FAOD are shown in Table 1.

In short, the clinical manifestations are produced by one or two mechanisms, alone or in combination: intoxication and energy deficit. As a consequence of the defect in the β-oxidation, an intracellular accumulation of fatty acids and their derivatives occurs, including the intermediate acyl-CoAs [11]. The types of acyl-CoAs accumulated depend on the nature of the enzymatic block. Macro- and microvesicular lipidic deposits are observed in liver, muscle and heart cells. A blockage in FAO leads to the ω-oxidation of fatty acids in microsomes, through cytochrome P450, with the production of dicarboxylic acids, which can be oxidized by β-oxidation and shortened [12].

Due to the deficiency of acetyl-CoA, the activation of gluconeogenesis, ureagenesis or ketogenesis are not possible. The resulting energy deficit can cause hypoketotic hypoglycemia, lactic acidemia or hyperammonemia [13].

The inability of the liver to metabolize the fatty acids results in hepatomegaly, liver dysfunction, steatosis, and a decreased production of ketones. Regarding the clinical manifestations of CPT I deficiency during pregnancy, heterozygous women with an affected fetus can develop a fatty liver. HELLP syndrome (hemolysis, elevated liver enzymes and low platelets) has been identified during pregnancies with fetuses affected by SCAD deficiency, and, in relation to hepatic steatosis, in pregnant women with MCAD-deficient fetuses, too. In the case of LCHAD-deficient fetuses, mainly those with the 1528G>C variant, up to 79% of pregnant women have been reported with clinical evidence of pre-eclampsia, acute fatty liver and HELLP syndrome. In fetuses affected by LCKAT, HELLP syndrome can also develop independently of phenotype [14].

Muscle and cardiac involvement is clear, considering the high requirement of energy that these tissues make of FAO. The accumulation of long-chain acylcarnitines can have a toxic effect in the phospholipids of the sarcolemma, depending on the chain length and the free carboxylic functional groups. These acylcarnitines interact with different ion channels and produce cardiac arrhythmias [15], which have not been observed in short- or medium-chain defects, as they do not require the carnitine system to enter the mitochondria.

**Figure 2:**
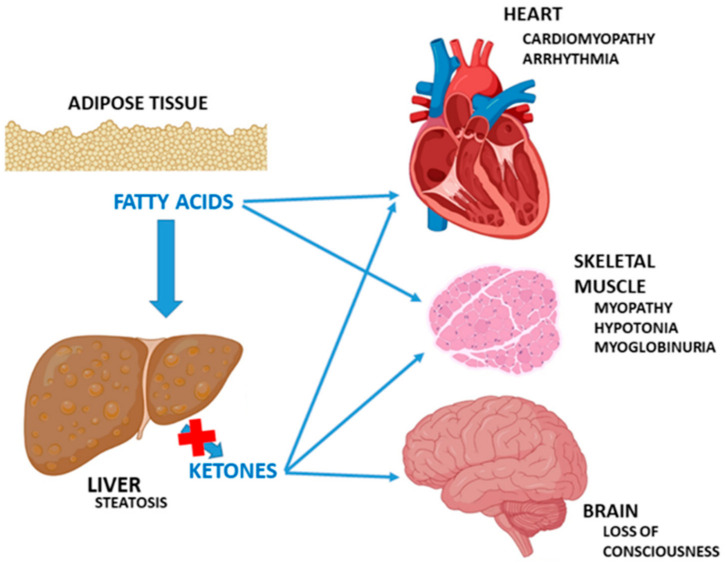
Defective fatty acid oxidation during fasting and metabolic stress. Adapted from Longo et al. [16] by BioRender.

Neurological involvement may be mediated by the effects of hypoglycemia, and by the toxic effects of the accumulation of fatty acids or their metabolites. Moreover, this autointoxication, similar to other organic acidemias, could explain the fulminant decompensation and sudden death seen in some patients [16]. Figure 2 graphically summarizes these consequences.

Several mechanisms have been suggested to explain the similarity of this condition to Reye’s Syndrome:

- Reduction in the catabolic capacity of FA to produce energy, especially in periods of fasting;
- No synthesis of ketone bodies, which exacerbates the energy deficit;
- Mitochondrial sequestration of coenzyme-A as a fatty acyl-CoA ester, leading to an intramitochondrial deficit of acetyl-CoA with effects on the tricarboxylic acid cycle, the synthesis of N-acetylglutamate, and other pathways;
- The toxic effect of the accumulation of the intermediates of acyl-CoA and its acylcarnitines;
- The toxicity of the increased concentration of free fatty acids. The carnitine transport system is potentially inhibited by medium- and long-chain acylcarnitines, and so the accumulaiton of these metabolites could explain the secondary carnitine deficit in most FAODs.

Initially, the prognoses of FAODs were considered poor. At present, the prognoses are favorable for the vast majority of patients, once treatment has been established, with significantly improved morbidity and mortality after newborn screening.

## 2. Study of Metabolites in the Diagnosis of Fatty Acid Oxidation Defects

The correct diagnosis of patients has been difficult for many years, since there has been a lack of adequate biomarkers in the blood, serum or plasma, and of enzymatic assays that allow the specification of a definite FAOD [17]. The use of tandem mass spectrometry at the end of the 20th century manifested great changes via improvements in acylcarnitine analysis, and quality control schemes are now well-established [18]. This biochemical method has now become the main choice for the diagnosis of FAODs, revolutionizing newborn screening with its implementation. However, other techniques have also demonstrated value in acylcarnitine analysis, such as GC-CI-MS or HPLC/fluorescence [19]. Nevertheless, some patients require more detailed studies to provide diagnosis when the biochemical, enzymatic or molecular studies’ results are unclear.

There are three main pillars of the biochemical study of FAODs [20,21]:

- Carnitine (total and free) and acylcarnitines tests in blood or plasma/serum:

The carnitine levels help us in diagnosis, both in the acute phase and under stability. Under conditions of the carnitine transport defect, blood/plasma levels are greatly decreased, with persistent urinary excretion. In CPT I, the total carnitine levels are elevated. In the rest of the FAODs, they are reduced by 25–50%, although they can be found within normal ranges. Acylcarnitines are specific to certain errors, both in decompensation and intercrisis. Carnitine and acylcarnitines are also attracting attention as biomarkers in other disorders related to neither FAODs [22] nor other metabolic diseases [23,24]. They are useful in the prognosis of diabetes, sepsis, cancer and heart failure. Therefore, these reductions should also be considered in cases of altered acylcarnitine plasma profiles, in addition to the suspicion of an inborn error in the metabolism.

The main source of carnitine is the diet in non-vegetarians, but it is also a product of an endogenous synthesis of lysine and methionine. These steps of carnitine biosynthesis are come before carnitine shuttle and fatty acid oxidation, and some metabolic disorders have been described [25,26], but not being an objective in this review.

- Urine analysis for organic acids and acylglycines:

Unsaturated medium-chain hypoketotic dicarboxylic aciduria (C8:1>C8, C10:1>C10) is found in MCAD (occasionally with ketonuria), VLCAD and LCHAD deficiencies. In addition, C6-C14 3-hydroxydicarboxylic aciduria is found in LCHAD.

Acylglycines are formed by the transesterification of an acyl-CoA ester with glycine through the action of acyl-CoA:glycine-N-acyltransferase (glycine-N-acylase). In SCAD, the urinary excretion of butyrylglycine (BG) is increased. A combination of the urinary acylglycines hexanolglycine (HG), phenylpropionylglycine (PPG) and suberglycine (SG) is specific to MCAD. In MAD, acylglycine excretion is variable, depending on the severity of the defect and the clinical conditions; in severe forms we see elevations in the glycines of both the straight and the branched chains; in moderate forms, only increases in BG, isobutyrylglycine (IBG) and isovalerylglycine (IVG) are found [27].

- Blood test for free fatty acids and 3-hydroxyacids.

Free fatty acids and 3-hydroxy-fatty acids can be determined simultaneously in plasma (normal values < 1 µmol/L) [28]. Significant elevations in the 3-hydroxy-C14 to C18 fatty acids, and in a lesser proportion C10 and C12, are found in LCHAD and MTP, in acute periods (in a higher proportion), under stable conditions, and during dietary treatment with low consumption of long-chain fatty acids, which is a relevant marker for both diagnosis and follow-up. During seizures, elevations in 3-hydroxy-medium-chain fatty acids are found in MCAD, VLCAD, MAD, CPT I and CPT II. There is a generalized but non-specific increase in long-chain free fatty acids in LCHAD and MTP. However, in MCAD, a characteristic strong increase in fatty acids C8, C10:1 and C10 (octanoic, decenoic and decanoic acids) is observed. Although C8 is the highest, C10:1 is very important for both the diagnosis and monitoring of MCAD, since the other two are less specific, and can be elevated if the patient ingests medium-chain triglycerides. In VLCAD, the characteristic increase is mainly of the long-chain fatty acids C14:1, C14:2 and C16:2. In MAD, both the neonatal form and the moderate form present a variable increase in the metabolites found in MCAD and VLCAD, with fatty acids C6 to C18 being higher in the neonatal form and C10:1 and C12:1 higher in the moderate form. Patients with CPT I and CPT II show an increase in long-chain fatty acids, mainly C16 and C18, although none are pathognomonic, while medium-chain fatty acids are found within normal ranges.

Table 2 enumerates the biochemical markers mentioned above.

During the stabilization phase, if the patient is not in a state of metabolic decompensation, a metabolic study should be performed to detect specific markers, which may remain elevated even during periods of stability [29].

In this way, the diagnosis of FAODs has been simplified by determining acylcarnitine levels in blood [30]. Since most FAODs are associated with alterations in carnitine metabolism, acylcarnitines are elevated in body fluids and tissues, both in periods of metabolic decompensation and in stable periods. Additionally, the determination of free fatty acids and 3-OH-fatty acids is also useful in this situation (Table 2).

When the results of the metabolite studies are not conclusive, the study of the oxidation of deuterated or [U-^13^C]-palmitate in fibroblasts, and the analysis of the acylcarnitines generated, are both recommended [31,32]. This method could reveal a metabolic alteration, regardless of the clinical status and treatment of the patient, and provide a characteristic acylcarnitine profile for each entity. Therefore, an oxidation study of the stable isotopically labeled palmitate is of great use to diagnostic confirmation, even in cases in which the initial study of metabolites has not been informative. The exceptions are CPT II/CACT and LCHAD/MTP deficiencies, which cannot be differentiated. In addition, this method is not useful in cases of carnitine transporter deficiency, so other study approaches, such as the incorporation of carnitine in fibroblasts, enzyme activity or molecular analysis, will be necessary to confirm the diagnosis.

### 2.1. Carnitine Transporter Deficiency

Carnitine transporter deficiency (CTD, OCTN2 deficiency) (OMIM 212140) should be considered as a primary carnitine deficiency to supply CPT I. An approximate incidence of 1:40,000 newborns has been estimated, which varies between countries, reaching 1:300 in the Faroe Islands [33]. The transporter is expressed in muscles, the heart, the kidneys, as well as the leukocytes and fibroblasts, and is not expressed in the liver, where carnitine enters by passive diffusion. At the cardiac and muscular level, due to a deficit in carnitine cell penetration, FAO is deficient. This process is aggravated by the low reabsorption of carnitine into the kidneys, which induces very low serum levels, decreasing passive hepatic diffusion and altering ketogenesis. The accumulated acyl-CoA substrates can be used for the synthesis of triglycerides, or can contribute to peroxisomal β-oxidation, which produces dicarboxylic acids that do not require carnitine to enter into the mitochondria, and are thus able to be completely oxidized at this level. This fact explains the low or non-existent levels of dicarboxylic acids in this inborn error of metabolism.

Diagnostic signs include hypoketotic hypoglycemia, which can be accompanied by hyperammonemia and hypertransaminasemia. Very low serum levels of carnitine (<10 µmol/L), the increased urinary excretion of carnitine and the low levels of relevant dicarboxylic aciduria, in patients with this symptomatology are practically pathognomonic of this condition (Table 2). The accumulation of acylcarnitines in the plasma and acylglycines in urine are not observed. Reduced levels of C2, C3, C16, C18 and C18:1 acylcarnitines in the plasma or blood are sometimes found. For biochemical detection and diagnosis, attention should be paid to various aspects, especially during newborn screening, when the possible patient is still asymptomatic. For example, false positives may be generated by carnitine deprivation, due to the mother’s vegetarian diet, parenteral nutrition, hemodialysis [34] other treatments, or the CTD deficiency of asymptomatic mothers [35]. Recent dried blood spots have also been reported to be the useful samples, with the discarding of those the stability of which is questionable, since the spontaneous hydrolysis of acylcarnitines could increase the levels of free carnitine in a sample [36].

Diagnosis will be confirmed using fibroblast or leukocyte culture [37,38,39], where defects in the plasma membrane of the carnitine transporter can be demonstrated, and/or by a genetic study. The clinical and biochemical response to carnitine treatment is significant, restoring the serum levels of carnitine to almost normal levels, along with the formation of hepatic ketogenesis and improved clinical signs of myopathy and cardiomyopathy [40], despite not restoring carnitine levels at the muscular or cardiac level.

Prenatal diagnosis can be made using amniotic fluid cells.

### 2.2. Carnitine Palmitoyltransferase I Deficit

Carnitine palmitoyltransferase I (CPT I) deficiency (OMIM 255120) is a serious disorder, which is expressed in the liver, kidneys and fibroblasts but not in muscles. The prevalence ranges from 1:500,000 to 1:1,000,000, with very high rates in the Alaskan native population (1:780), Canada, Greenland and northeast Siberia [8]. Due to enzymatic deficit, acylcarnitines are not formed, so long-chain substrates cannot penetrate the mitochondria for oxidation. The alternative metabolic pathway produces medium-chain intermediates, which are subsequently oxidized by the mitochondria.

Diagnosis is facilitated by hypoketotic hypoglycemia and moderate hyperammonemia [41,42]. Some patients have elevated CPK levels and metabolic acidosis, attributable to renal tubular acidosis during acute episodes.

Biochemical diagnosis is permitted by the elevation of free carnitine levels in the serum, plasma or dried blood spots, which distinguishes CPT I deficiency from other FAODs (Table 2). Sometimes, short-chain acylcarnitines levels can also be increased, and long-chain acylcarnitines decreased [29]. Normal levels of free carnitine have been frequently reported in genetically confirmed patients; therefore, the use of ratios (C0/(C16 + C18), C3/C16, (C16 + C18:1)/C2) has been extended to confirm biochemical diagnoses and to avoid false negatives [43]. The low levels of long-chain acylcarnitines in plasma or serum expected in CPT I-deficient patients are barely observable, since normal levels here are lower than those in blood [44], making it necessary to use these ratios.

Dicarboxylic aciduria, with the elevation of mainly C12 dicarboxylic acid and 3-hydroxyglutaric acid, could be present [45].

Definitive diagnoses are made by measuring the enzymatic activity in fibroblasts or leukocytes [46], and/or by genetic study.

### 2.3. Carnitine-Acylcarnitine Translocase Deficit

Carnitine-acylcarnitine translocase deficiency (CACTD) (OMIM 212138) is a severe FAO defect, in which long-chain acylcarnitines accumulate outside the mitochondrial matrix due to enzyme deficiency. Some of the acylcarnitines are probably degraded in peroxisomes, with short-chain acylcarnitines being observed in urine.

#### Diagnosis

CACT deficiency is presented with hypoketotic hypoglycemia and severe dicarboxylic aciduria with an excess of unsaturated species. Under these conditions, free carnitinemia is very low (<5 µmol/L), and the levels of long-chain acylcarnitines (C16, C18 and C18:1) are high, similar to those seen with CPT II deficiency (Table 2) [30,47,48]. Diagnosis is confirmed by demonstrating enzymatic deficit, and/or by a genetic study of the fibroblasts or the lymphocyte culture. Prenatal diagnosis can be made in amniocytes or chorionic villi via enzyme assay or molecular analysis.

### 2.4. Carnitine Palmitoyltransferase II Deficit

Carnitine palmitoyltransferase II (CPT II) deficiency (OMIM 255110) is a FAO defect with a variable spectrum of symptoms and severity (from death to more moderate conditions). The incidence is estimated to be between 1:750,000 and 1:2,000,000, with more than 300 patients identified to date.

Due to the CPT II deficiency, long-chain acylcarnitines cannot be converted into their corresponding acyl-CoAs, thus accumulating in the mitochondrial matrix. These acylcarnitines can be transported outside the mitochondria, establishing the alternative metabolic pathway of peroxisomal ω-oxidation with the production of medium-chain intermediates, which are subsequently completely oxidized in the mitochondria and do not require the carnitine cycle. This is why dicarboxylic aciduria is not seen in this entity.

#### Diagnosis

Frequently, hyperammonemia, metabolic acidosis, hypoketotic hypoglycemia and elevated levels of CPK are observed, not appreciating dicarboxylic aciduria. Plasma free and total carnitine levels are greatly reduced in the hepatocardiomuscular form of the condition, unlike CPT I. At the muscular level, carnitine levels are not altered, or are slightly decreased. Characteristically, C16, C18 and C18: 1 acylcarnitines are elevated [49], similar to that seen in CACT [50] (Table 2), and the ratio of (C16 + C18:1)/C2 is also increased. However, in milder forms, normal acylcarnitine levels and a normal ratio cannot always preclude CPT II; false negatives of the myopathic adult-onset form have been reported after newborn screening [51], ratio in dried blood spots could be normal more frequently than in plasma/serum [44]. The diagnosis will be confirmed by measurement of the enzymatic activity [52,53] in fibroblasts or leukocytes (or in the skeletal muscle and liver), and/or by genetic study [54].

Prenatal diagnosis can be made via mutational or enzymatic studies of chorionic villi or amniocytes. Antenatal manifestations can occur, such as cerebral dysgenesis and Dandy–Walker malformations. The results of the acylcarnitine analysis of amniotic fluid are not sufficiently altered to ground a prenatal diagnosis [55].

### 2.5. Short-Chain Acyl-CoA Dehydrogenase Deficiency

Short-chain Acyl-CoA dehydrogenase deficiency (SCADD) (OMIM 201470) is a FAOD with an estimated frequency from 1:50,000 to at least 1:1000 in some countries (The Netherlands [56,57]), or to 1:100 in some ethnic groups (the Roma group in Slovakia [58]).

#### Diagnosis

In this condition, unlike in other FAODs, the absence of hypoketotic hypoglycemia is noteworthy: SCAD is required only at the end of the β-oxidation cycle, the previous steps are sufficient stimulus for gluconeogenesis and ketogenesis. Carnitinemia is present at normal levels. However, the ratio of esterified carnitine/free carnitine is increased.

**Figure 3:**
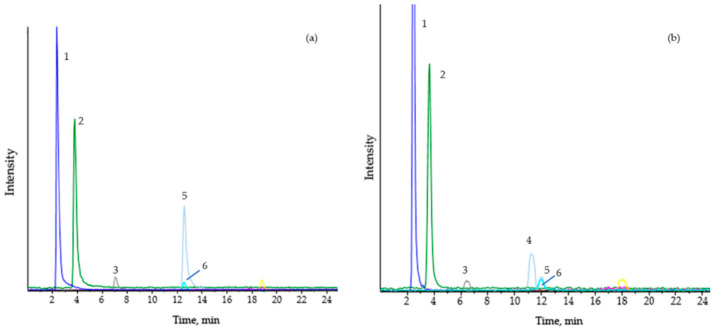
Analysis of short-chain acylcarnitines isomers in serum by HPLC/MS/MS as described by Ferrer et al. (2007) [59]. (**a**) Short-chain acyl-CoA dehydrogenase deficiency. (**b**) Isobutyryl-CoA dehydrogenase deficiency. (1) C0, free carnitine; (2) C2, acetylcarnitine; (3) C3, propionylcarnitine; (4) C4, isobutyrylcarnitine; (5) C4, butyrylcarnitine; (6) d3-C4, d3-butyrylcarnitine (internal standard).

The elevation in butyrylcarnitine (C4) levels in plasma is characteristic. Considering C4 is usually analyzed by FIA-MS/MS (flow injection analysis–tandem mass spectrometry) in newborn screening programs, and chromatographic separation is not used, C4 could also correspond to isobutyrylcarnitine, a biomarker of isobutyryl-CoA dehydrogenase deficiency (OMIM 611238) in the valine metabolism. Therefore, in the absence of other initial biochemical data, increased levels of C4 in the dried blood spots or plasma of patients should be reanalyzed, such as by chromatographic separation online (Figure 3).

In urinary organic acid analysis, diagnosis is grounded on the increased excretion of ethylmalonic acid (EMA) (>20 mmol/mol) and methylsuccinic acid (the hydrolyzed product of the isomerization of ethylmalonyl-CoA by methylmalonyl-CoA mutase), as well as butyrylglycine (C4-glycine); although these products are intermittently eliminated [60]. They are only usually elevated in acute episodes, and are normal in inter-crisis periods. However, EMA is also a typical sign of multiple acyl-CoA dehydrogenase deficiency, mitochondrial respiratory chain disorders, ethylmalonic encephalopathy syndrome, the intramitochondrial flavin adenine dinucleotide pathway and the vomiting disease from Jamaica, in treatments with ifosfasmide, and has even been associated with a polymorphism (G209S) in the SCAD protein [61]. Persistent lactic acidemia has been found in some patients. Definitive diagnosis must be made via the measurement of enzymatic activity in the fibroblast, lymphocyte or muscle culture [62], by in vitro fatty acid oxidation studies [63] and/or by genetic study.

### 2.6. Medium-Chain Acyl-CoA Dehydrogenase Deficiency

Medium-chain acyl-CoA dehydrogenase (MCAD) deficiency (OMIM 201450) is the most common disorder in FAO. Its incidence is approximately 1:10,000 Caucasian newborns, ranging from 1:6400 to 1:460,000 [3]. Following newborn screening, an incidence of 1:10,000–1:18,000 has been estimated. In the general population, the frequency of heterozygotes is 1–2%. It has been described more frequently in northern Europe and North America, although with the introduction of newborn screening programs in more countries, its detection rate has increased.

Due to the enzymatic block, 4 to 12 carbon atom fatty acids cannot be metabolized, leading to an increase in medium-chain acyl-CoA esters, medium-chain fatty acids, dicarboxylic acids, acylglycines and medium-chain acylcarnitines in plasma and urine [64]. Secondarily, systemic carnitine deficiency complicates the FAO.

#### Diagnosis

The analytical findings may include:

1. In plasma, hypoglycemia, the elevation of free fatty acids C8 (octanoic acid), C10:1 (4-*cis*-decenoic acid) and C10 (decanoic acid). Additionally, 4-*cis*-Decenoic acid is very specific to this condition. Low levels of free carnitine are found (10–50% of normal). The acylcarnitine/free carnitine ratio is increased. The levels of acylcarnitines in plasma are usually high. The following acylcarnitines are unique and specific to this condition: C6; 4-*cis*-C8:1; 5-*cis*-C8:1; C8 and 4-*cis*-C10:1. They are present both in periods of decompensation (especially C8) and in periods of stability, as well as before the debut, so they can be used for newborn metabolic screening or in postmortem studies. There is a correlation between genotype and acylcarnitine levels, with an overlap in increased concentrations of C8 in carriers and affected with moderate levels [65,66]. In these cases, enzyme activity assays and very careful genetic studies could be used for diagnosis, mostly via newborn screening [67]. The typical and diagnostic values found in MCAD are as follows:

Octanoylcarnitine (C8) greater than 0.3 µmol/L, C8/C10 ratio greater than 5. C8/C2 ratio greater than 0.1. Normal to low levels of acylcarnitines shorter than C6 and longer than C10. An unknown C8:1-interfering compound has been reported to cause false positive cases in newborn screening analyses, because it falsely increases C8 levels; therefore, calculation of the ratio C8/C8:1 is recommended [68].

Acylglycines can also be analyzed and detected at increased levels in dried blood spots. This fact can be exploited in newborn screening to improve the detection of different FAODs and other inborn errors of metabolism. In MCAD patients, suberylglycine and hexanoylglycine are detected, and their levels can reduce the false positive rate [69].

Hyperammonemia, increased liver enzymes, hyperuricemia and elevated CPK are usually found during acute attacks.

2. In the urine, levels of free carnitine are usually reduced, with a relative increase in acylcarnitines. The acylcarnitine/free carnitine ratio is increased. However, the diagnostic value of acylcarnitines in urine is limited, since they can vary and be detected in other FAODs.

Dicarboxylic acids (C6-C12), as adipic, suberic, dehydrosuberic (octenedioic), sebacic, dehydrosebacic (decenedioic), 3-hydroxysebacic and dodecanedioic acids, could be excreted at increased levels.

The excretion of corresponding hydroxyacids by ω-oxidation, 5-hydroxyhexanoic and 7-hydroxyoctanoic acids could also be increased. This dicarboxylic aciduria is not specific to MCAD, and can be found in other FAODs, in diabetic ketoacidosis, and after the administration of medium-chain triglycerides (MCT).

Glycine conjugates, hexanolglycine, suberglycine and phenylpropionylglycine could be very highly excreted during seizures, but also in their classic form during clinical stability; however, the two first acylglycines are also found in MADD. Butyrylglycine elevation appears in MADD and not in MCAD, so the two conditions can be distinguished. Phenylpropionylglycine is a specific marker for MCAD, although it may not be detected if the patient is undergoing antibiotic therapy, or in neonates and young infants, since it is an intestinal bacterial product. New tandem mass spectrometric methods are very useful for increasing the detection and recovery of this acylglycine [70]. Acylglycines are also excreted following MCT supplementation, especially suberylglycine [27,71]. However, this dicarboxylic acid profile and the pathological pattern can easily be differentiated, since, under MCT, sebacic acid is excreted at higher levels than suberic and adipic acids.

Ketone bodies are found in low levels, or are absent. Although hypoketotic hypoglycemia is a common finding, ketone body production could occur in episodes of decompensation, so the finding of hypoglycemia with ketonuria should not exclude the diagnosis of MCAD. In a published series, up to 29% of patients with MCAD had ketonuria [72].

The study of octanoyl-CoA oxidation in lymphocytes is currently the most efficient and reliable way to differentiate the functional effects of the mutant genotype; activity less than or equal to 20% confirms the disease, between 20 and 30% would require supervision and control, while symptomatic disease is not present if the activity is higher than 30% [73].

Conclusive diagnosis is achieved via the determination of the enzymatic activity in fibroblasts, lymphocytes or tissues (liver, heart, skeletal muscle and amniocytes), and/or by genetic study.

Prenatal diagnosis can be achieved when the enzyme defect in amniocytes is demonstrated. Other possibilities include the in vitro testing of FAO and the determination of the genetic variation in amniocytes.

The detection of MCAD has been included in newborn screening, given its relative frequency (similar to PKU); a fatal course and failure in the neonatal period can generally be controlled with dietary measures, and diagnosis in dried blood spots can be achieved in the first days of life through routine blood sampling for the detection as well as other inherited metabolic diseases. Other strategies proposed for newborn screening include the detection of the prevalent A985G variation, 4-*cis*-decenoic acid or hexanolglycine [74]. Levels of MCAD-specific acylcarnitines are higher in the neonatal period than in other stages of life.

### 2.7. 3-Hydroxyacyl-CoA Dehydrogenase Deficiency

The 3-Hydroxyacyl-CoA dehydrogenase (HAD) deficiency (OMIM 231530, 609975), previously referred to as short- and medium-chain 3-hydroxyacyl-CoA dehydrogenase (M/SCHAD) deficiency, is a heterogeneously presenting FAOD. This dehydrogenase catalyzes the oxidation of 3-hydroxyacyl-CoA C4-C10 substrates. At present, it should be classified as a congenital hyperinsulinism syndrome [6]. Its prevalence is less than 1:1,000,000.

#### Diagnosis

There are C6-C14 dicarboxylic aciduria and 3-hydroxydicarboxylic aciduria. In both types, the acylcarnitine/free carnitine ratio is high. Neither acylglycines nor alterations in free fatty or 3-hydroxy-long-chain fatty acids are found. C4-hydroxyacylcarnitine levels are elevated in plasma (Table 2). The elevated excretion of 3-hydroxyglutaric acid is very characteristic of this defect. This metabolite is a well-known biomarker of glutaric aciduria type I; however, clinical data, normal levels of glutarylcarnitine and a normal excretion of glutaric acid point to an HAD defect [75]. Hyperketonuria is also observed in these patients. The diagnosis is confirmed by genetic study.

### 2.8. Mitochondrial Trifunctional Protein and Long-Chain 3-Hydroxyacyl-CoA Dehydrogenase Deficiencies

Biochemically, two forms of deficiency have been described:

In the first form (Type I), the three activities of the MTP (OMIM 609015) are deficient, with an estimated incidence after newborn screening of 1:750,000. In the second form (Type II), isolated dehydrogenase activity deficiency (LCHAD) is observed (OMIM 609016), along with normal hydratase activity (78%) and moderately decreased thiolase activity (59% of the control). It has an incidence after newborn screening of 1:250,000.

For both forms (LCHAD/MTP), the prevalence is 0.41 per 100,000 (1:244,000) [8,76].

#### Diagnosis

Clinically, the presence of retinopathy and peripheral neuropathy linked to 3-OH-dicarboxylic aciduria help to differentiate these deficiencies from other FAODs. Hepatomegaly is a common finding, although it can be present in other errors found in this group of diseases, as well as in other clinical manifestations, such as dilated or hypertrophic cardiomyopathy, which can be very severe but reversed with the appropriate treatment [77].

In metabolic decompensations, the following findings are almost constant in plasma:

- Hypoglycemia, the elevation of aminotransferases and CPK, hyperammonemia and secondary carnitine deficiency. Lactic acidosis is often moderate or severe (it has been suggested that pyruvate oxidation is compromised by the toxicity of acyl-CoA esters and their long-chain 3-hydroxyacyl-CoAs, while not occurring in medium-chain defects);
- The elevation of 3-hydroxyacylcarnitines C14-C18, OH-C16:0, OH-C18:2 and OH-C18:1 being characteristic [78];
- The elevation of free fatty acids C14 to C18 and 3-OH-fatty acids C14 to C18, which are also increased in periods of stability and under dietary treatment.

Anemia that can be present in many patients.

In urine, the findings include C6-C14 dicarboxylic aciduria, the absence of ketonuria, the absence of acylglycines and the increased excretion of C6-C14 3-OH-dicarboxylic acids in acute episodes (Table 2).

Definitive diagnosis must be made by measuring the enzymatic activity in fibroblasts or lymphocytes [79], and/or by genetic and biochemical studies. The primary defects of the respiratory chain can be presented with nonketotic hypoglycemia, dicarboxylic aciduria and/or dicarboxylic 3-hydroxyaciduria, simulating LCHAD, although low carnitine concentrations and hypoglycemia are not characteristic of respiratory chain defects.

Regarding the isolated short-chain enoyl-CoA hydratase deficiency, characteristic biomarkers the analysis of organic acids, acylcarnitines and cysteine and cysteamine derivatives arise from a blockage in the valine catabolic pathway [80,81], despite also being considered a FAOD [11].

### 2.9. Long-Chain 3-Ketoacyl-CoA Thiolase Deficiency

Within the MTP, only the β-subunit (HADHB) is affected, and a deficit in long-chain 3-ketoacyl-coenzyme A thiolase (LCKAT) (OMIM 143450) occurs, which is a rare FAOD with few patients identified to date.

#### Diagnosis

Dicarboxylic aciduria and hydroxy-dicarboxylic aciduria have been observed. Carnitine levels are found at the lower limits of normality. Elevations in C14:1 and C16:1 acylcarnitines, and C16-OH and C18:1-OH hydroxyacylcarnitines, have been observed [82]. Diagnosis is confirmed via a culture of lymphocytes and fibroblasts, and/or by a genetic study.

### 2.10. Very Long-Chain Acyl-CoA Dehydrogenase Deficiency

Very long-chain acyl-CoA dehydrogenase (VLCAD) is one of the four acyl-CoA dehydrogenases with different chain length specificities, and it catalyzes the first stage of FAO. Its deficiency (VLCADD) (OMIM 201475) is a FAOD that has recently been molecularly characterized, and earlier clinical descriptions have been categorized as LCAD. Newborn screening has reported a high incidence, of 1/31,500 [83,84].

#### Diagnosis

The findings in plasma include an increase in free fatty acids C14:1 n-9 (5-*cis*-tetradecenoic acid) and lower proportions of C14:2 and C16:2 as specific markers of VLCAD deficiency. However, moderate elevations in these markers, associated with other abnormal fatty acids, have been observed in MAD and LCHAD deficiencies. These metabolites remain elevated even during periods of metabolic stabilization. The corresponding C14:1 acylcarnitine (tetradecenoylcarnitine) is also specific to this entity [85]. Other long-chain acylcarnitine species (C14, C16, C16:1, C16:2, C18:0, C18:1 and C18:2) are also elevated. A secondary free carnitine deficit is also seen.

Lactic acidosis is found, as in LCHAD deficiency. It has been suggested that pyruvate oxidation is compromised in these patients by the toxicity of acyl-CoA esters and the long-chain 3-hydroxyacyl-CoA, which do not occur in medium-chain defects.

In newborn screening, false positives have been reported in patients, following a loss of body weight and other circumstances of catalysis of fatty acids [86] and fasting [87]. Therefore, poor milk supply, poor sucking, and other causes of body weight loss should be reviewed in these situations. This possible overdiagnosis when detecting VLCAD deficiency makes necessary the use of ratios (C14:1/C12:1, C14:1/C16, C14:1/C2) [88] (Table 2), as well as enzymatic [89] and genetic analyses, to complete the final diagnosis in asymptomatic patients undergoing newborn screening [90]. In addition, it may be true that these acylcarnitines are elevated under conditions of VLCAD deficiency or in fasting periods, but other metabolites clearly differentiate these two conditions. Fasting individuals show elevations in free fatty and dicarboxylic acids, as well as ketone bodies, in their plasma [87].

As a novel approach to the diagnosis of VLCAD deficiencies, untargeted metabolomic strategies are growing in importance. New biomarkers have been reported and described to prognosticate the severity of the deficiency [91]. These markers include acylcarnitines C18:2 and C20, as well as dihydroretinol and deoxycytidine monophosphate, the levels of which increase in the severe form, and which could be more informative than C14:1.

**Figure 4:**
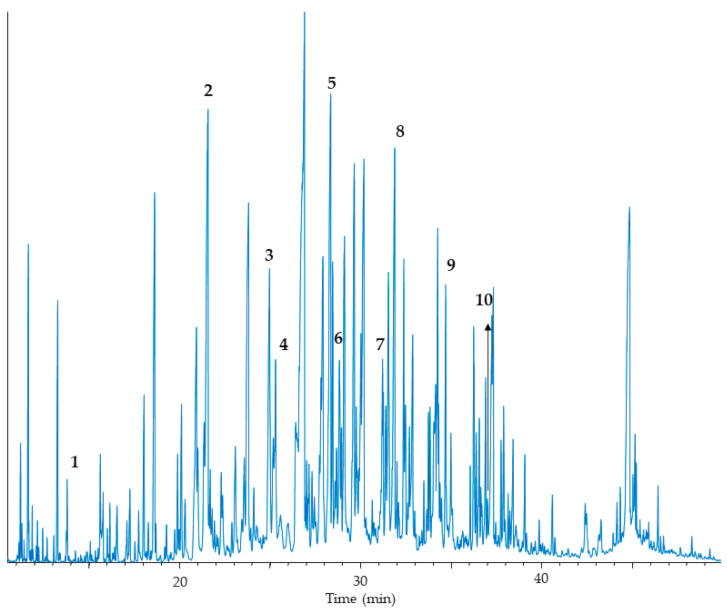
Urinary organic acid analysis in a VLCAD patient by GC/MS. Very slightly increased excretion of (1) 3-hydroxybutyric. Highly increased excretion of (2) adipic, (3) dehydrosuberic, (4) suberic, (5) dehydrosebacic, (6) sebacic, (7) 3-hydroxydecenedioic, (8) 3-hydroxydecanedioic, (9) 3-hydroxydodecanedioic and (10) 3-hydroxytetradecenedioic. Arrow indicates the location of peak 10.

In urine, ketonuria is scarcely observed. C6-C14-saturated, but also unsaturated dicarboxylic aciduria is found during periods of metabolic decompensation, as well as 3-hydroxydicarboxylic aciduria (Figure 4). The excreted levels of acylglycines and acylcarnitines are not increased, but there is a low level of excretion of carnitine (Table 2).

Diagnosis will be confirmed by determination of the enzymatic activity in fibroblasts, lymphocytes or tissues (it is expressed in the liver, heart and skeletal muscles), by oxidation flux studies, which can also predict the severity [92], and/or by genetic study, which can be complicated, given the wide mutational spectrum and the lack of a prevalent mutation.

Prenatal diagnosis can be achieved in amniocytes or chorionic villi.

Acyl-CoA dehydrogenase 9 (*ACAD9*) plays a role in complex I in the mitochondrial respiratory chain, but also has a dehydrogenase effect on 14- to 20-carbon fatty acids, similarly to VLCAD [11,93]. However, these two enzymes are involved in different physiologic functions, and cannot compensate for each other’s deficiencies. ACAD9 deficiency (OMIM 611126) could manifest no abnormalities or reductions in the free and total carnitine levels in plasma [94], with correlated losses in the activity and severity of the disease [95], but highly increased ketonuria, in contrast to VLCADD.

### 2.11. 2,4-Dienoyl-CoA Reductase Deficiency

2,4-Dienoyl-CoA reductase (DECR1) deficiency (OMIM 222745) was first described by Charles Roe et al. in 1990 [96].

This enzyme is necessary for the breakdown of even-chain double-bonded unsaturated fatty acids, such as linoleic acid. It converts 2,4-dienoyl-CoA to 3-*trans*-enoyl-CoA. It has been included in some newborn screening programs, although it is of uncertain clinical significance, similarly to SCAD.

#### Diagnosis

The diagnosis is grounded on the presence in plasma and urine of 2-*trans*-4-*cis*-C10:2 acylcarnitine (decadienoylcarnitine) [96], which is an intermediate in the degradation of linoleic acid and is a substrate for 2,4-dienoyl-CoA reductase. Hypoglycemia, dicarboxylic aciduria and acylglycines are not observed. The free fatty acid levels are normal, with low or normal carnitine levels and a high acylcarnitine/free carnitine ratio (Table 2).

The diagnosis is confirmed by genetic studies.

### 2.12. Medium-Chain 3-Ketoacyl-CoA Thiolase Deficiency

Medium-chain 3-ketoacyl-CoA thiolase deficiency (MCKAT) (OMIM 602199) is a FAOD that was described by Kamijo T et al. in 1997 [97]. This enzyme catalyzes the medium- to short-chain cleavage of 3-ketoacyl-CoAs.

#### Diagnosis

The organic acids present in urine can indicate ketotic lactic aciduria with C6-C12 dicarboxylic aciduria (especially C10-C12) (Table 2).

This diagnosis is confirmed by the determination of the enzymatic activity in fibroblasts.

### 2.13. Long-Chain Fatty Acid Transport Defect

The long-chain fatty acid transporter is located at the plasmatic membrane level, and its defect (DTAGCL) (OMIM 603376) was described in two patients in 1998 by Al-Odaib et al. [98].

#### Diagnosis

Acylcarnitines, acylglycines and organic aciduria are not observed at abnormal levels. Plasma free carnitine is found to be normal or decreased, with a normal free acylcarnitine/carnitine ratio (Table 2).

### 2.14. Multiple Acyl-CoA Dehydrogenase Deficiency

Multiple acyl-CoA dehydrogenase (MAD) deficiency (glutaric aciduria type II) (OMIM 231680) can be caused by mutations in at least three of the genes involved in the mitochondrial ETF/ETF-QO complex *(ETFA*, *ETFB* and *ETFDH*), a pathway that transfers electrons from the first stage of β-oxidation to the electron transport system. These enzymatic deficits block the β-oxidation of fatty acids, as well as the oxidation of branched-chain amino acids and glutaryl-CoA in the metabolic pathway of lysine, tryptophan and hydroxylysine. A prevalence of 1:200,000 is estimated, which is variable between countries [99].

It can be detected by expanded newborn screening.

#### Diagnosis

The biochemical findings from plasma include hypoketotic hypoglycemia, acidosis with increased anion gap, and an increase in free fatty acid levels. Both the neonatal form and the moderate form manifest a variable increase in the metabolites found in MCAD or VLCAD, with the levels of C6 to C18 fatty acids being higher in the neonatal and the late-onset mild form [100], and C10:1 and C12:1 being higher in the moderate form.

The analysis of acylcarntines can show secondary free carnitine deficiency, and the elevation of C4 and C5 to 18-carbon atom acylcarnitines. In plasma analyses with very low free carnitine levels, the profile of acylcarnitines can be falsely identified as normal. Therefore, the analysis should be repeated after carnitine supplementation [101,102]. Serum acylcarnitine analysis allows for a better diagnosis of late-onset MAD deficiency compared to other MADD biomarkers, such as DBS acylcarnitines and the organic acids in urine, abnormal levels of which are not always clear [103].

Defects in riboflavin intake or transport and FAD synthesis or transport can resemble a MAD deficiency [14,17]. The identification of these disorders could be included in the value of acylcarnitines analysis, since FAD acts as a cofactor in MAD, and the resulting acylcarnitine profiles in serum are very similar [104].

In urine, MAD deficiency presents an absence of ketonuria and an increase in ethylmalonic, glutaric and isovaleric acids excretion. The excretion of acylglycines is variable, depending on the severity of the defect and the clinical conditions of the patient. Severe forms show an elevation in the acylglycines of both straight and branched chains. In moderate forms, only increases in butyryl- (BG), isobutyryl- (IBG) and isovalerylglycine (IVG) are found, while these may be normal in the asymptomatic phase (Table 2).

Diagnosis is confirmed by measuring the level of the electron-transporting flavoprotein in fibroblasts, and/or by genetic study.

## 3. Conclusions

In summary, the knowledge about and study of biomarkers of FAODs in patients suspected of harboring them is a powerful tool for the characterization of these disorders. Some of these disorders can be identified more rapidly in a single determination, but others require combinations of different procedures. In this review, we also highlight differences between types of samples and patient conditions. Concerning newborn screening programs, new experience is being gained since new biochemical profiles are detected, different from the classical, and which must be differentiated from carriers or false positives. Molecular analyses can finally confirm clinical and/or biochemical suspicions, or determine the genetic cause in inconclusive cases. However, and more after NBS implementation, it is being proved the increasing need of studies in biomarkers and biochemical results along with genetics in the solving of challenges related to FAODs.

## Figures and Tables

**Table 1:** Clinical manifestations in mitochondrial fatty acid and carnitine system diseases.

Deficiency (Protein Abbr.)	Year Description	Mckusick OMIM	Fasting Hypoglycemia	Ketonuria	Myocardiopathy	Myopathy	Hepatopathy	Others
Fatty acid transport (FATP1)	1998	600691	+ *	− **	−	−	+++ ***	
Long-chain fatty acid transport (DTAGCL)	1998	603376	+	−	−	−	+++	
Acyl-CoA synthase (LACS)	1990	152425						
Carnitine transport (OCTN2)	1975	212140	+	−	+++	+	−	Cardiomegaly Lipid vacuoles in muscle Sudden death
Carnitine palmitoyltrasnferase I, hepatic (CPT IA)	1981	255120 600528	+	−	−	+	+++	Renal tubular acidosis Fatty liver Sudden death
Carnitine palmitoyltrasnferase I, muscular (CPT IB)	1996	601987	No patients reported, up to now. Lethal murine model					
Carnitine palmitoyltrasnferase I, cerebral (CPT IC)	2002	608846						
Carnitine acylcarnitine translocase (CACT)	1992	212138	+++	−	+++	+	+++	Severe neonatal onset Cardiomegaly Bradycardia. Arrythmia Sudden death
Carnitine palmitoyltrasnferase II (CPT II), Severe neonatal	1988	608836	+++	−	+++	+	+++	Renal cysts Lipid vacuoles in muscle, heart, liver, kidney
Carnitine palmitoyltrasnferase II (CPT II), Infant–adult onset	1973	255110 600649	−	−	−	+++	−	Myoglobinuria Rhabdomyolysis Weakness and exercise-induced muscle soreness
Very long-chain acyl-CoA dehydrogenase (VLCAD)	1993	201475	+	−	+++	+	+++	Sudden death Myoglobinuria Rhabdomyolysis
Medium-chain acyl-CoA dehydrogenase (MCAD)	1982	201450	+++	−/+	−	−	+++	Sudden death
Short-chain acyl-CoA dehydrogenase (SCAD)	1984	201470	+	−/+	+	+	+	Growth delay Ophthalmoplegia Sudden death No symptoms
Long-chain 3-hydroxyacyl-CoA dehydrogenase (LCHAD)	1988	609016	+	−	+++	+	+++	Myoglobinuria Retinopathy Peripheral polyneuropathy Sudden death
Mitochondrial trifunctional protein (MTP)	1992	609015	+	−	+++	+	+++	Myoglobinuria Retinopathy Peripheral polyneuropathy Sudden death
3-Hydroxyacyl-CoA dehydrogenase (HAD)	2001	231530 609975	+++	−	−	+	−	Hyperinsulinism Mental delay Seizures Sudden death
Medium-chain 3-ketoacyl-CoA thiolase (MCKAT)	1997	602199	+	−	+	+	+++	Rhabdomyolysis Neonatal myoglobinuria Sudden death
Multiple acyl-CoA dehydrogenase, (MAD) ETFDH	1985	231680 231675	+++	−	+	+	+	Congenital malformations
Multiple acyl-CoA dehydrogenase (MAD) ETF-α	1985	231680	+++	−	+	+	+	Congenital malformations
Multiple acyl-CoA dehydrogenase (MAD) ETF-β	1990	231680 130410	+++	−	+	+	+	Congenital malformations
2,4-Dienoyl-CoA reductase (DECR1)	1990	222745	−	−	−	+++	−	Hyperlysinemia Growth delay Microcephaly Ventriculomegaly Cerebellar atrophy Seizures
Long-chain 3-ketoacyl-CoA thiolase (LCKAT)	2006	143450	+	−	+++	−	−	Cardiomyopathy

* +: present; ** −: absent; *** +++: severe.

**Table 2:** Biochemical markers and chromosomal location in mitochondrial ß-oxidation and carnitine system defects.

Deficiency	Gen	Locus	Free Carnitine (Plasma)	Acylcarnitines (Plasma or Dried Blood Spots)		Free Fatty Acids/ 3-Hydroxyacids (Plasma)	Organic Acids (Urine)	Acylglycines (Urine)
				Elevated	Decreased			
Carnitine Transporter (CTD)	*SLC22A5*	5q31.1	**very low, <10% controls ^1^**	No elevations	**C2**, C3, **C16**, C18, C18:1	Normal	Normal	Normal
Carnitine palmitoyltransferase I (CPT I)	*CPT1A*	11q13.3	**high**	Ratios: C3/C16, **C0/(C16 + C18)**	C16, C18, C18:1, C18:2 Ratios: **(C16 + C18:1)/C2**	3-OH-medium chain C16-C18	C12 dicarboxylics	Normal
Carnitine acylcarnitine translocase (CACT)	*SLC25A20*	3p21.31	decreased	C14, **C16**, **C16:1**, C16-DC, **C18**, **C18:1**, **C18:2**, C18:1-DC Ratios: C14:1/C2, **(C16 + C18:1)/C2**	C2 Ratios: C3/C16, **C0/(C16 + C18)**	Normal	Severe dicarboxylic aciduria unsaturated dicarboxylic excess	Normal
Carnitine palmitoyltransferase II (CPT II)	*CPT2*	1p32.3	decreased	C6-DC, C8-DC, C12, C14, **C16**, **C16:1**, C16-DC, **C18**, **C18:1**, **C18:2**, C18:1-DC Ratios: C14:1/C2, **(C16 + C18:1)/C2**	C2 Ratios: C3/C16, **C0/(C16 + C18)**	Normal or 3-OH-medium chain C16-C18	Normal	Normal
Very long-chain acyl-CoA dehydrogenase (VLCAD)	*ACADVL*	17p13.1	decreased	C12, C12:1, **C14**, **C14:1, C14:2**, C16, C16:1, C18, C18:1, C18:2 ratios: **C14:1/C2**, C14:1/C12:1, **C14:1/C16**	No decreases	**3-OH-medium chain** **C14:1n-9, C14:2, C16:2**	Dicarboxylic acids C6 to C14	Normal
Medium-chain acyl-CoA dehydrogenase (MCAD)	*ACADM*	1p31.1	decreased	C6, **C8**, C3-DC, C5-DC, C10, C10:1 Ratios: **C8/C2**, **C8/C10**, C8/C16 C8/C8:1	C2	**3-OH-medium chain** **C8, C10** **C10:1n-6**	Dicarboxylic acids C6 to C12, 5-OH-hexanoic, 7-OH-octanoic acids	C6 to C8 **Hexanoylglycine Suberylglycine Phenylpropionylglycine**
Short-chain acyl-CoA dehydrogenase (SCAD)	*ACADS*	12q24.31	normal	**C4** Ratios: C4/C2, C4/C3, C4/C8	No decreases	Normal	Ethylmalonic, methylsuccinic acids	Butyryglycine
3-hydroxyacyl-CoA dehydrogenase (LCHAD)	*HADHA*	2p23.3	decreased	C14, C14:1, C14:2, C14-OH, C14:1-OH, C16:1, **C16-OH**, **C16:1-OH**, C18, C18:1, C18:2, **C18-OH, C18:1-OH,** **C18:2-OH** Ratios: C14:1/C2, C14:1/C16, **C16-OH/C16**, C18-OH/C18	No decreases	C14-C18 Long-chain 3-OH-acids (COH14:0, **COH14:1;n-9**, COH18:0)	OH-dicarboxylic acids C6 to C14 Dicarboxylic acids C6 to C14	Normal
Mitochondrial trifunctional protein (MTP)	*HADHA* *HADHB*	2p23.3 2p23.3	decreased	C14, C14:1, C14:2, C16:1, **C16-OH,** **C16:1-OH**, C18-OH, C18:1-OH, C18:2-OH Ratios: C14:1/C2, C14:1/C16, **C16-OH/C16**, C18-OH/C18	No decreases	**C14:1n-9**/ Long-chain 3-OH-acids (C14 to C18) C14-C18 acids	OH-dicarboxylic acids C6 to C14 Dicarboxylic acids C6 to C14	Normal
3-hydroxyacyl-CoA dehydrogenase (HAD)	*HADH*	4q25	decreased	**C4-OH, C6-OH** Ratios: C4-OH/C4	No decreases	Normal	Dicarboxylic and OH-dicarboxylic acids C6 to C14 **3-OH-glutaric acid**	Normal
Multiple acyl-CoA dehydrogenase (ETF-QO, α-ETF, β-ETF) (MAD)	*ETFDH* *ETFA* *ETFB*	4q32.1 15q24.2 19q13.41	decreased	**C4**, **C5**, C5-DC, **C6**, **C8**, C10:1, **C10**, **C12**, C12:1, **C14**, C14:1, C14:2, **C16**, C16:1 Ratios: C4/C2, C4/C3, C5/C0, C5/C2, C5/C3, **C8/C2**, **C14:1/C2**, C14:1/C16, (C16 + C18:1)/C2	No decreases	**C10:1n-6, C14:1n-9**	Dicarboxylic acid, ethylmalonic, glutaric and 2-OH-glutaric acids	Butyryl-, isobutyryl, **isovaleryl**, 2-metehylbutyryl-, tyglyl-, **hexanoyl**- and **suberylglycines**
2,4-dienoyl-CoA reductase (DECR1)	*NADK2*	5p13.2	decreased	**C10:2**	No decreases	Normal	Normal	Normal Hyperlysinemia
Long-chain 3-ketoacyl-CoA thiolase (LCKAT)	*HADHB*	2p23.3	lower normal limits	C14:1 and C16:1 C16OH and C18:1OH.	No decreases		Dicarboxylic and OH-dicarboxylic acids	¿ ^2^
MCKAT	*MCKAT*	¿	¿	¿	¿	Normal	Dicarboxylic and OH-dicarboxylic acids (specially C10–C12)	Normal
DTAGCL	*DTAGCL*	¿	Normal/decreased	No abnormality	No decreases	¿	Normal	Normal

^1^ More relevant abnormalities in bold. ^2^ ¿:Unknown, not reported.
